# Prevalence, knowledge and factors associated with e-cigarette use among parents of secondary school children

**DOI:** 10.1016/j.puhip.2022.100334

**Published:** 2022-11-02

**Authors:** Julie Doherty, Jenny Davison, Marian McLaughlin, Melanie Giles, Lynn Dunwoody, Claire McDowell, Sarah Butter, Elaine Wilmont, Ellen Simpson

**Affiliations:** a Psychology Research Institute, Ulster University, Northern Ireland, UK; b Public Health Agency for Northern Ireland, Belfast, Northern Ireland, UK

**Keywords:** e-cigarettes, Vaping, Theory of planned behavior, Parents, Attitudes, Adolescents

## Abstract

**Objectives:**

Identify prevalence rates and attitudes towards e-cigarette use among parents to inform prevention strategies designed to reduce uptake in young people.

**Study design:**

A mixed methods sequential study guided by the Theory of Planned Behaviour.

**Methods:**

This research involved two phases. Phase one was an elicitation study using focus groups, interviews and open-ended questionnaires (N = 17) to elicit parental behavioural, normative, and control beliefs around e-cigarette use. Findings from phase 1 were used to inform a questionnaire administered to a sample of 612 parents in phase 2. The aim of phase 2 was to identify and explain factors that influence parental attitudes and motivations towards e-cigarette use. Parents were recruited through post-primary schools and were sent a link to an online survey.

**Results:**

Approximately 19% of parents had tried an e-cigarette, with 9% reporting current use. Sociodemographic variables, TPB constructs and knowledge of e-cigarettes, accounted for 43% and 60% of ever use and intention to use an e-cigarette, respectively. Intention, gender, age and free school meal entitlement were associated with ever use. Intention to use an e-cigarette was related to lower educational level, current smoking of traditional cigarettes, more positive attitudes, greater social pressure, having greater control over use and knowledge.

**Conclusions:**

Prevention strategies designed to reduce uptake in young people should raise awareness of the health risks of e-cigarette use, legislation and regulations and highlight the role parents play in encouraging young people to abstain from using an e-cigarette.

## 1 Introduction

E-cigarette use continues to increase globally in adults and adolescents. Throughout Europe, there are approximately 7.6 million e-cigarette users [1], and of that, there are 3.6 million in Great Britain [2]. Current trends are generating diverse views and debate on their public health implications. For example, those advocating a harm reduction approach to tobacco control propose that switching traditional smokers to e-cigarettes has the potential to reduce harm and contribute to public health agendas. This conclusion is based on literature reporting positive health outcomes of switching [3]. However, others oppose this, reporting numerous health implications, including asthma exacerbation; poorer respiratory function; lung disease and throat and mouth irritation [[4], [5], [6]]. However, there is widespread agreement that uptake of e-cigarette use among young people should be prevented.

Social context has been shown to influence risk behaviours [7,8]. Research has found that parental exposure is a key predictor of uptake in alcohol use, tobacco smoking, and adolescent e-cigarette use [[9], [10], [11], [12], [13], [14], [15]]. Findings from a longitudinal cohort study found young people aged 11–12 years old were five times more susceptible to e-cigarette use if a family member used one [11]. Further, adolescent e-cigarette use was influenced by their perception of parental attitudes [12]. Moore et al. [13] examined primary school children's (*N* = 2218) perceptions of both electronic and tobacco cigarettes and associations with parental smoking and vaping. The results showed susceptibility to vaping was higher than smoking, however, viewing an e-cigarette as a stop-smoking aid reduced susceptibility.

The Theory of Planned Behaviour (TPB) was used as the framework for this research [15]. According to the theory, behaviour is determined by intentions to perform a behaviour. Intention is further influenced by three constructs, attitudes (advantages and disadvantages of engaging in a behaviour), subjective norms (SN – social influences on behaviour) and perceived behavioural control (PBC – facilitators and barriers to a behaviour). The TPB has been used extensively to identify factors that influence intentions and explain the underlying cognitive processes towards smoking and to look at quitting behaviour [[16], [17], [18]]. More recently, the TPB has been used to identify factors that influence e-cigarette use in adolescents and young adults [[19], [20], [21], [22]]. Using a shorter version of the theory, Trumbo and Kim found that factors predicting students intention to use an e-cigarette were positive responses to adverts and the belief that they were less addictive [19]. A recent survey of college undergraduates examined e-cigarette use using the TPB. Findings identified attitudes and norms as the main factors that influence use. The results suggest uptake in students could be reduced by challenging positive attitudes and norms [20]. Similarly, Wang [21] examined use and perceptions of e-cigarettes and identified attitudes and subjective norms as the main predictors of intention. While these studies provide support for the TPB as a theoretical framework, none of this research has used the TPB to examine factors that influence parents’ intention to use an e-cigarette, even though they may influence use in their children. This study addresses this gap, and the findings will help inform preventative interventions aimed at reducing e-cigarette use among adolescents.

The aim of the current study was to use an extended version of the TPB to determine, knowledge, prevalence of use and beliefs among parents of adolescents (11–16 years) around e-cigarette use. The objectives were: 1. to determine the salient attitudinal, social, and behavioural factors surrounding e-cigarette use; 2. to construct and administer a TPB questionnaire; 3. to determine knowledge and identify predictors of e-cigarette use in parents of adolescents.

## 2 Methods

Consistent with the TPB, a sequential exploratory design utilising qualitative and quantitative methods was employed [23,24]. Phase 1 was a qualitative elicitation study to identify the salient beliefs around parental e-cigarette use [25]. Phase 2 was a TPB survey based on the most prevalent salient beliefs, identified in Phase 1.

## 3 Phase 1: Elicitation study

### 3.1 Participants

Ethical approval was obtained from the University Research Ethics Committee (UREC), in accordance with The Code of Ethics of the World Medical Association (Declaration of Helsinki). Parents of adolescents aged 11–16 years, were recruited through five post-primary schools in Northern Ireland (NI), one from each education authority. Four schools replied and two agreed to participate. Expression of interest forms were sent home with pupils (from one randomly selected class per year group, 5 classes in total). Those returning a form were contacted by phone to arrange interviews or focus groups, if a suitable time was not found, open-ended questionnaires were posted out. Parents, both males and females, smokers and non-smokers, and e-cigarette users and non-users were eligible to take part, purposeful recruitment ensured these groups were represented. Recruitment continued until data saturation was reached [26]. This resulted in five telephone interviews and two focus groups, the former lasting around 20 min and the latter lasting from 30 to 40 min. Four individuals completed open-ended questionnaires.

### 3.2 Materials and procedure

Consistent with previous research, three approaches to data collection (focus groups, interviews, and open-ended questionnaires) were used to aid recruitment and enhance the validity of the data through triangulation [27,28]. Focus groups were held on school premises, interviews conducted by phone and open-ended questionnaires completed and returned to the researcher either in person or via post. Discussions and open-ended questionnaires asked about awareness of e-cigarettes, their use as a stop-smoking device, and a gateway to smoking. In keeping with the TPB, participants were asked about the advantages/disadvantages of using an e-cigarette (behavioural beliefs); people who would approve/disapprove of using an e-cigarette (normative beliefs) and enablers/barriers to using an e-cigarette (control beliefs) [23,24]. Participants completed a short questionnaire collecting socio-demographic information (e.g., age, sex, and educational level), smoking behaviour, and e-cigarette use. Focus groups and interviews were audio recorded and transcribed verbatim prior to analyses, the open-ended questionnaires were reviewed and analysed.

### 3.3 Data analyses

Thematic and summative data analyses were used following the procedures outlined by Francis et al. [24,29] Transcripts were read several times to ensure familiarity with the data. Beliefs were identified, labelled, and assigned to one of the TPB constructs. Summative analysis determined the most frequently occurring beliefs within each construct. Beliefs were rank ordered and the top 75% used to formulate belief-based items investigated further in Phase 2. Two researchers carried out this process independently (ES & JDa) to increase reliability. Differences in coding were discussed, and a consensus agreed.

## 4 Results

### 4.1 Sample description

Seventeen parents (male: *n* = 3; female: *n* = 14; age range 33–48 years; *M*^*age*^ = 41 years) took part in phase 1, with 17% identifying as a current e-cigarette user and 28% as a tobacco smoker, and 50% reporting tertiary level education.

### 4.2 Parents’ behavioural, normative and control beliefs about e-cigarettes

All TPB constructs were relevant to e-cigarette use, outcomes with exemplar quotes are presented in supplementary file 1.

***Behavioural beliefs***. Participants identified an e-cigarette as a smoking cessation device and nicotine management system. However, some were concerned that these devices were used as an alternative and not as a stop smoking aid. Many were unsure of the health impact of using an e-cigarette but believed switching may benefit smokers. When compared to tobacco smoking, e-cigarettes were considered cheaper, more pleasant smelling, providing a similar smoking experience and more socially acceptable.

***Normative beliefs***. In relation to approval to use e-cigarettes, reference was made to the social influence of partners, family members and friends. More specifically, that these reference groups would disapprove of e-cigarette use among non-smokers but would approve of tobacco smokers switching to an e-cigarette.

***Control beliefs***. Facilitators and barriers centred around social and environmental factors. The actions of others and accessibility were believed to support use. The variety of flavours and affordability were also identified as facilitating factors. However, some smokers suggested the variety of flavours was not a key determinant in their decision-making. Barriers included limiting access to products and banning use in specific areas, as well as the unknown long-term health implications.

## 5 Phase 2: Questionnaire development and survey

### 5.1 Participants

The inclusion criteria outlined in Phase 1 was applied in Phase 2. Recruitment was facilitated through post-primary schools in NI. Participating schools were asked to send home an information sheet with pupils (up to two classes per year group) and/or distribute a link to a questionnaire via the schools’ electronic communication systems, inviting their parents/guardians to take part.

### 5.2 Questionnaire development and procedure

The questionnaire had five sections, including socio-demographic information, e-cigarette use and experimentation, direct and indirect measures of the TPB constructs, smoking behaviour and knowledge of e-cigarettes. E-cigarette and tobacco items were developed through consultation with key stakeholders to support comparisons across studies. The TPB items were informed by Phase 1 and constructed in line with guidance [21]. Knowledge items were based on the literature and respondents indicated if each was true or false. Information on the number of items included in each section, sample questions and response formats are provided in supplementary file 2.

The questionnaire was piloted (*N* = 16) and feedback obtained, no issues were identified. Responses from the pilot sample were included in the final analyses. The questionnaire was primarily completed using Qualtrics, a small number (n = 4) of parents opted to complete a paper version.

### 5.3 Data analyses

SPSS vs 25 was used to analyse the data. Mean scores were computed for each of the direct measures of the TPB constructs. Higher mean scores indicated more favourable attitudes, higher levels of perceived social pressure and perceptions of control. Prior to computing mean scores, the internal consistency of items measuring the direct TPB constructs were assessed using Cronbach's Alpha (see supplementary file 2).

Indirect measures were constructed using a two-part formulation. Each attitudinal, subjective and control belief scale were scored 1 to 5 and multiplied by the corresponding evaluation, compliance, and power scale items (scored −2 to +2) [24]. The products were summed for each subscale. Knowledge items answered correctly were scored 1, higher scores indicating a higher level of knowledge.

Preliminary analyses including checks for missing data, homogeneity of variance and internal consistency of scales. Skewness (+2 to −2), kurtosis values (<10) and internal reliability scores were within an acceptable range α=>0.6 [24]. Homogeneity scores (below 0.05) and variance inflation factor (VIF) and tolerance statistic for TPB variables were acceptable (less than 10) [30]. Descriptive statistics provided information on participant characteristics. Two distinct Pearson's Bivariate correlations were performed to explore the relationship between the TPB constructs in both non-users and ever users. Hierarchical linear and logistic regression analyses were conducted to identify and explain factors that influence intention and ever use, respectively.

## 6 Results

### 6.1 Sample characteristics

Sample characteristics are presented in Table 1. In total, 612 parents (*M*_age_ = 43.72 years; *SD =* 6.09) were recruited. The majority were female, employed/self-employed and had a third level education. Most parents were not in receipt of free school meals (FSM), which was included as a social indicator of deprivation.

**Table 1:** Characteristics of the study sample.

Variable	N	%
Sex		
Female	530	86.6
Male	81	13.2
Prefer not to say	1	0.2
**Highest level of education**		
Primary/secondary	283	46.3
Tertiary	323	52.8
Missing	6	1.0
**Child in receipt of free school meals**		
Yes	100	16.3
No	511	83.5
Missing	1	0.2
**Employment status**		
Employed/self-employed	484	79.1
Unemployed	112	18.3
Student	1	0.2
Retired	6	1.0
Other	3	0.5
Missing	6	1.0

### 6.2 Prevalence of use (e-cigarette and tobacco smoking)

Approximately 19% of the sample reported using an e-cigarette, of these, 9% identified as a current user and 10% as an ever user. Nearly 89% of current users reported everyday use, with over half indicating they intended to stop using an e-cigarette in the future (54%), 32% do not. Weekly spend on e-cigarette products was low, for most (61%), less than £5 each week. In relation to tobacco smoking, approximately 50% of participants had used tobacco cigarettes, 21% of these were current users. 29% of past smokers had used an e-cigarette, with 82% continuing to use the device.

### 6.3 Knowledge of E-Cigarettes

Most believed e-cigarettes were addictive (82%), that they contain nicotine (77%), were cheaper than cigarettes (71%) and that the legal age to purchase was 18 years (69%). Just over half believed e-cigarettes did not produce tar and carbon monoxide (53%), 33% said e-cigarettes were regulated and licenced and 23% believed they were 95% less harmful than tobacco cigarettes.

### 6.4 Predicting ever use of, and intentions to use, e-cigarettes

Pearson's Bivariate correlation analyses were computed for ever users and non-users, to explore the relationships between study variables, prior to conducting the regression analyses, see Table 2. Correlations ranged from small to medium and were in the expected direction.

**Table 2:** Pearson's bivariate correlations of the TPB variables and knowledge for ever users of EC (above diagonal) and never users of EC (below diagonal).

Study variables	1	2	3	4	5	6	7	8	9
1. Intention	1	**.**597**	.336**	.274**	−.154	.644**	.474**	.583**	.446**
2. Attitude	**.**195*	1	.495**	.245**	.024	.530**	.593**	.473**	.278**
3. SN	.249**	.217**	1	.059	−.115	.296**	.696**	.243**	.042
4. Self-efficacy	−.048	.049	−.091	1	.193*	.276**	.164	.343**	.215**
5. PBC	−.172**	−.184**	−.267**	.242**	1	−.045	−.251*	.006	−.342**
6. Composite value of attitude	.037	.351**	.049	.205**	−.054	1	.399**	.490**	.304**
7. Composite value of SN	.221**	.200**	.454**	−.046	−.111*	.030	1	.361**	.170
8. Composite value of PBC	.071	.058	.112*	.115*	.019	.068	.024	1	.265**
9. Knowledge of EC	.018	.108*	.031	.121*	−.036**	.169**	−.006	.022	1

**Note**: SN = subjective norm, PBC = perceived behavioural control, EC = electronic cigarettes. **p = 0.01 (2-tailed), *p = 0.05 (2-tailed).

To determine predictors of ever use of e-cigarettes (dependent variable) a hierarchical logistic regression analysis was conducted. Socio-demographic variables (age, sex, FSM uptake, level of education) entered in step 1 accounted for 7% (Cox & Snell R Square) of the variance (*χ*^2^ = 40.657, df = 4, *p* < 0.001) in ever use. When direct measures of the TPB constructs were added in step two, the model accounted for approximately 36% of the variance (*χ*^2^ = 190.560, df = 4, *p* < 0.001), increasing to 43% with the addition of intention (*χ*^2^ = 69.579, df = 1, *p* < 0.001). The final step included knowledge but did not add to the variance in ever use (*χ*^2^ = 0.908, df = 1, *p* = 0.341). As shown in Table 3, being male (β = 1.127, *p* < 0.05), FSM uptake (β = 1.004, *p* < 0.05), age (β = −0.105, *p* ≤ 0.001), attitude (β = 0.510, *p* < 0.05), SN (β = 0.670, *p* < 0.01), self-efficacy (β = 0.426, *p* < 0.05), PBC (β = 0.579, *p* < 0.05) and intention (β = 1.592, *p* ≤ 0.001) were associated with ever use of e-cigarette. The odds ratios suggested the most influential predictors was intention, being male, SN and FSM entitlement with increases in these variables leading to someone being nearly 5 times or 3 times (for the latter three variables) more likely to have tried them.

**Table 3:** Outcome of the final step in the hierarchical logistic regression analysis.

							95% CI	
Variables	β	S.E.	Wald	Df	*p*	Exp(B)	Lower	Upper
Male	1.127	.444	6.460	1	**.011**	3.087	1.294	7.364
Tertiary	−.321	.391	.673	1	.412	.725	.337	1.562
FSM	1.004	.472	4.532	1	**.033**	2.730	1.083	6.881
Age	−.105	.031	11.484	1	**.001**	.901	.848	.957
Attitude	.510	.253	4.061	1	**.044**	1.665	1.014	2.733
SN	.670	.220	9.310	1	**.002**	3.005	1.271	3.005
Self-efficacy	.426	.169	6.345	1	**.012**	1.531	1.099	2.132
PBC	.579	.294	3.862	1	**.049**	1.784	1.002	3.177
Intention	1.592	.303	27.538	1	**.000**	4.914	2.711	8.906
Knowledge	.175	.185	.896	1	.344	1.191	.829	1.712

**Note**: SN = subjective norm, PBC = perceived behavioural control, FSM = free school meals, CI = confidence intervals. Reference groups in analysis: sex = female, level of education = primary/secondary. Significant *p* values in bold.

A hierarchical linear regression analysis was performed to identify the factors associated with intention to use an e-cigarette, see Table 4. Intention was related to age, level of education and FSM entitlement in step 1 of the model, accounting for almost 5% of the variance. There was a significant change in R^2^ with the addition of tobacco smoking (ever use) in step 2, accounting for almost 17% of the variance. The direct measures of the TPB constructs were added in step 3, explaining 47% of the variance which increased to 59% with indirect measures of the TPB in step 4. Knowledge explained 1% of the variance when added in step 5. In the final step, those who reported tertiary level education scored lower on intention to use (β = −.246, *p* < 0.001; spc [2] = 0.0259) while higher intention was associated with having smoked tobacco cigarettes (β = 0.257, *p* < 0.001; spc [2] = 0.0278), more favourable attitudes (β = 0.255, *p* < 0.001; spc [2] = 0.0506), increased social pressure (β = 0.105, *p* < 0.05; spc [2] = 0.0090), attitudinal beliefs (β = 0.009, *p* < 0.001; spc [2] = 0.0445), beliefs about the views of significant others (β = 0.012, p < 0.001; spc [2] = 0.04), control beliefs (β = 0.030, *p* < 0.001; spc [2] = 0.1204) and knowledge (β = 0.102, *p* < 0.01; spc [2] = 0.0225). The unique contribution of each variable was established by calculating the semi-partial correlation squared. Intentions being influenced by lower level of education, ever tobacco smoking, direct and indirect measures of attitude, greater social pressure, control beliefs and knowledge about e-cigarettes.

**Table 4:** Hierarchical regression analyses with intention as the dependent variable and socio-demographics, ever smoked, TPB variables and knowledge as independent variables.

Step	Predictor variables	R [2]	ΔR^2^	F Change (df)	*p*
1	Socio-demographic variables	.047	.039	F(4,517) = 6.316	<0.001
2	Ever smoked	.167	.159	F(1, 516) = 74.582	<0.001
3	TPB variables	.475	.466	F(4, 512) = 75.003	<0.001
4	Indirect TPB measures	.591	.581	F(3, 503) = 48.099	<0.001
5	Knowledge	.600	.590	F(1, 508) = 11.629	<0.01

**Note**. SN = subjective norm, PBC = perceived behavioural control, FSM = free school meals, TPB = Theory of Planned Behaviour. Reference groups in analysis: sex = female, level of education = primary/secondary.

### 6.5 Explaining intentions to use e-cigarettes

To gain a better understanding of what specific attitudinal, social and behavioural beliefs influenced intentions to use e-cigarettes, three distinct regression analyses were performed. Behavioural beliefs accounted for 27% of the variance in intention (F(9, 595) = 25.011, p < 0.001), with a cheaper alternative to traditional cigarettes (β = 0.040, *p* < 0.001; spc [2] = 0.0213), preventing the smell associated with tobacco smoking (β = 0.037, *p* < 0.05; spc [2] = 0.0108), managing a nicotine addiction (β = 0.032, *p* < 0.05; spc [2] = 0.006) and provide a similar experience to traditional cigarettes (β = 0.065, *p* < 0.001; spc [2] = 0.036) found to directly influence intention to use. Normative beliefs explained 30% of the variance in intention (F(4, 599) = 65.172, p < 0.001). One's friends were the only normative reference to make a unique contribution to intention (β = 0.077, *p* < 0.001; spc [2] = 0.022). Control beliefs accounted for nearly 50% of the variance in intention (F(8, 5523) = 64.716, p < 0.001). The variety of flavours (β = −0.171, *p* < 0.001; spc [2] = 0.048), owning an e-cigarette (β = 0.155, *p* < 0.001; spc [2] = 0.064) and convenience of purchasing e-cigarettes (β = 0.261, *p* < 0.001; spc [2] = 0.041) made a unique contribution to intention.

## 7 Discussion

Young people obtain important information about health behaviours from their parents, including smoking type behaviours [14]. Therefore, understanding e-cigarette use, attitudes and factors that influence use in parents has important implications for preventing uptake in young people. The current research used an extended version of the TPB to build on existing research and describe e-cigarette use among parents of adolescents. The findings showed that 19% of parents had used an e-cigarette previously, this is lower than rates reported in other studies [13,31]. Current use in this sample (9%) was slightly higher than among adults in Great Britain [2]. In keeping with the TPB, the study variables explained more of the variance in intention (60%) than ever use (43%) [31,32]. Ever use and intentions to use e-cigarettes were both predicted by more positive attitudes, greater social pressure and being in control.

Additional predictors of ever use were being younger, male, FSM entitlement, and for intentions, smoking cigarettes and knowledge of e-cigarettes, suggesting those from lower socio-economic groups may be more susceptible to using e-cigarettes.

The main facilitators and barriers related to intention to use an e-cigarette centred around control beliefs (50% of the variance), supporting previous research [33]. Two influences emerged as both facilitators and barriers to use in parents, these were owning a device and convenience of purchasing liquids, consistent with previous research [34,35]. If parents view e-cigarettes more positively, own a device and a choice of flavours, this could promote accessibility and facilitate experimentation in their children [12,36]. E-cigarette use in adolescents has been associated with family and peer smoking or using an e-cigarette and poor parental supervision [37,38]. Other research suggests it is peers that exert the greatest influence on behaviour of adolescents and not parental smoking [39,40]. For parents, the greatest social influence on e-cigarette use came from friends, consistent with previous research [34]. The link between subjective norm and intention is not surprising given that e-cigarettes were considered more socially acceptable in our elicitation study, such a view could be passed on to children and facilitate uptake.

In the current cohort, levels of use and experimentation with e-cigarettes were low and may be attributed to negative attitudes, with parents expressing concerns surrounding their health impact, nicotine maintenance and not cessation [41]. If parents perceive e-cigarettes as harmful, this has been associated with lower intentions to try them in their children [39]. However, parents who had more positive attitudes to e-cigarettes were more likely to plan to use them. Positive attitudes were related to them having the same effect, and being cheaper, than traditional cigarettes. Occasional use of e-cigarettes has been reported to enhance feelings of control and a desensitisation to risk of nicotine addiction [42]. This could have implications for health and well-being with regards maintaining or encouraging a nicotine addiction that may lead to smoking traditional cigarettes. There are strong links between using an e-cigarette and smoking, with dual use commonly reported. In the current study dual use was low with only 15% of current smokers reporting to use an e-cigarette regularly [2,43]. Knowledge was associated with intention to use an e-cigarette. A need for additional information on health risks for non-smokers, regulations, and licencing of these products, which are less well understood in parents was highlighted in the current study.

It is important to raise awareness in parents of the role they play as “active agents of change” in shaping their children's behaviour, through their own example of not using e-cigarettes and holding negative attitudes to their use [40]. Parents should encourage their children to abstain from use as parental attitudes were found to influence use [39]. Parents that use an e-cigarette may be providing opportunities for their children to experiment with such devices by creating environments where smoking type behaviours are acceptable.

## 8 Strengths and limitations

This is one of the first studies that employed an extended version of the TPB, as a theoretical framework, in a sequential mixed methods design to define parental use and beliefs about e-cigarettes. Furthermore, the data gathered from phase one and two of the research has identified the underlying processes influencing e-cigarette use in parents, addressing a paucity of research. Although this study represents an advance in our understanding of parental e-cigarette use, it is not without certain limitations. First, parents were sampled in NI where e-cigarette use for smoking cessation is not advocated by public health organisations, contrasting with other parts of the UK, which may have impacted attitudes towards use. Second, most participants reported tertiary level education, which may have impacted the findings.

## 9 Conclusions

Prevention strategies designed to reduce uptake among young people should include an educational intervention which raises parents' awareness and understanding of the health risks of e-cigarette use particularly for non-smokers, regulations and licencing and address the topic of using an e-cigarette as a stop-smoking device. The intervention should focus on delivering practical information that highlights how parents can play a role in discouraging use in young people. For example, sharing appropriate information with their children or minimising exposure. An educational intervention that targets these areas will impact parents' attitudes, knowledge and understanding of e-cigarette use. It would provide practical information on how to talk to young people about e-cigarettes and address potential control issues around ease of access. It is also important to consider how additional behaviour support could increase quit rates for those who have switched to use an e-cigarette. In turn, reducing young people's exposure and evidencing their role in smoking cessation rather than a new form of smoking.

## Declaration of competing interest

The authors declare that they have no known competing financial interests or personal relationships that could have appeared to influence the work reported in this paper.
