# Supplementary material for: Prevalence, knowledge and factors associated with e-cigarette use among parents of secondary school children
Source: Public Health Pract (Oxf). 2022 Nov 2;4:100334. doi: 10.1016/j.puhip.2022.100334 (PMC9664552; doi:10.1016/j.puhip.2022.100334)
Supplement: Multimedia component 1 [file mmc1.docx]

**Supplementary file 1**

*Salient beliefs about e-cigarettes with exemplar quotes*

| **Component** | **Theme** | **Salient beliefs** | **Exemplar quotes** |
| --- | --- | --- | --- |
| Attitude  (Behavioural Beliefs) | Stopping smoking | EC use can help people to stop smoking  EC use helps reduce smoking related anxiety/stress in smokers | “*People using them to come off cigarettes*”.  “*E-cigarettes are known for smoking cessation purposes*”.  “*To relax you and reduce anxiety*”.  “*Relieve stress*”. |
|  | Cost | EC use is cheaper than tobacco smoking | “*We have noticed a saving since my husband has been using one. There is an initial cost of buying the e-cigarette and then after you are just buying the liquid”*  “*Well from my mum’s point of view it was to sort of help reduce the use of cigarettes and to reduce the costs*”. |
|  | Appealing | No unpleasant smell with EC | “*With somebody who smokes cigarettes you always smell the cigarettes on them whereas with an e-cigarette the vapour seems to vaporise quite quickly”.*  “*You don’t stink*”. |
|  | Health | EC use is healthier than tobacco smoking | “*…they are certainly advertised as the healthier alternative*”.  “*Well I would expect to feel healthier in terms of the chest and wheezing and coughing…because of the reduction of the tar and the carbon monoxide and things*”. |
|  | Addiction | Maintains addiction to nicotine  EC use provides a similar experience as tobacco smoking  An EC is not as strong as tobacco cigarettes | “*I know my husband would say he is addicted…. now with being on the e-cigarette he is using that to feed his addiction, his habit*”.  “*They’re still equally as addictive with the nicotine in them”.*  “*Well it’ll give the body it’s nicotine craving*”.  “*Similar to cigarettes*”.  “*….it didn’t satisfy her addiction to the cigarettes*”. |
|  | Safety | EC have the potential to be a safer alternative to smoking | “*We were always afraid of her burning something , she suffers from sleep insomnia and can end up overtired and were always worried of her doing that when a cigarette was lit. At least with the e-cigarette we know that can’t happen so we feel happy in that sense. It has taken the worry away*”. |
| Subjective Norm (Normative Beliefs) | Partners | My partner | “*My partner he would rather I smoke an e-cigarette than smoke a cigarette*”.  “*Partner and family, I’m sure they would respect my decision*”. |
|  | Family | My family | “*My immediate family*”.  “*If its what I want to do I’m sure my family would support me*”.  “*Children*”. |
|  | Friends | My friends | “*Friends*”.  “*…family and friends*”. |
| Perceived Behavioural Control  (Control Beliefs**)** | Affordability | Being able to afford to maintain use | “*Money, affording one and affording to keep up the habit*”.  “*Money, affording to buy and continue using an e-cigarette*”. |
|  | Appeal | The variety of flavours | “*The one’s that I know that smoke them all they all enjoy the different flavours of them all like menthol or cherry or cola or something, you know there’s always a flavour to it. Whereas cigarettes are just smoke*”. |
|  | Availability | Convenience of use  Owning an EC | “*They are very accessible, more so now…I would say in the last 12 months there are shops/stores popping up everywhere selling them*”.  *“Just charge them and away you go, you don’t need to take a lighter with you or to be carrying your box and looking to see how many you have left”.*  *“There has to be a want to use or try an e-cigarette, you have to own one”.*  “…*you can very easily use this without anybody knowing you’re using it*”. |
|  | Risk | Unknown long-term health effects | “*I personally don’t think they’ve been tested long enough to know what would happen you know, cigarettes weren’t tested when they came out and now everyone knows how bad they are for you*”.  “*I’m really on the fence with it. Don’t get me wrong, it’s great to not be smoking cigarettes, but how great are these things, do we know*”. |
|  |  | Accessibility | “*Knowing where to buy one.*  *Getting an e-cigarette”.* |
|  | Conformity | Being in the company of EC users | “*It depends on the company they’re with*”.  “*If you were out and in the company of others using them*”. |

^Abbreviations: EC = electronic cigarette^
