# Supplementary material for: Prevalence, knowledge and factors associated with e-cigarette use among parents of secondary school children
Source: Public Health Pract (Oxf). 2022 Nov 2;4:100334. doi: 10.1016/j.puhip.2022.100334 (PMC9664552; doi:10.1016/j.puhip.2022.100334)
Supplement: Multimedia component 2 [file mmc2.docx]

**Supplementary file 2**

*Questionnaire items and response formats*

| **Section** | **N** | **Questionnaire items** |
| --- | --- | --- |
| **1.** Socio-demographic information | 5 | Age; sex; level of education: primary, secondary tertiary level; employment status: employed/self-employed, unemployed, student, retired, other; does your child receive free school meals: yes/no. |
| **2.** EC use | 5 | Have you ever used an EC/Do you currently use an EC: yes/no. EC users were asked; What is your weekly spend on EC products: less than £5; £5-£10; £10-£20; more than £20; Do you intend to stop using an EC: yes, within the next 6 months; sometime in the future; no, I have no plans to stop. How often do you use an EC: everyday; 4-6 times a week; 2-3 times a week; once a week; once a fortnight; less than once a month; Do you think EC should be promoted as a device to stop smoking: yes/no. |
| **3.** TPB constructs | 3 | **Intention:** I want/I intend/I am going to use an EC in the next month; (1) strongly disagree – (5) strongly agree. Cronbach’s **α** = .988. |
|  | 4 | **Direct attitude:** Using an EC in the next month would be: (1) harmful / (5) beneficial; (1) unhealthy / (5) healthy; (1) bad / (5) good; (1) foolish / (5) wise. Cronbach’s **α =** .933. |
|  | 18 | **Indirect attitude:** Behavioural beliefs (N = 9), e.g. ‘*Using an e-cigarette is a cheaper alternative to smoking’: (1)* unlikely – (5) likely and respective outcome evaluations (N = 9), e.g., ‘*A cheaper alternative to smoking is’*: bad (-2) - good (+2). |
|  | 3 | **Direct subjective norm:** People who are important to me think I should/people who are important to me want me to use an EC within the next month.; I feel under social pressure to use and EC within the next month: (1) strongly disagree - (5) strongly agree. Cronbach’s **α** = .903. |
|  | 8 | **Indirect subjective norm:** Motivations to comply (N = 4), e.g. ‘*Doing what my partner thinks matters to me*’: (-2) disagree – (+2) agree and respective normative beliefs (N = 4), e.g., ‘*My partner would approve of me using an e-cigarette’*: (1) unlikely - (5) likely. |
|  | 6 | **Direct perceived behavioural control (n=3):** The decision to use an EC in the next month is beyond my control: (1) strongly agree – (5) strongly disagree. Whether I use an EC in the next month is entirely up to me; It is mostly up to me if I use an EC or not within the next month: (1) strongly disagree – (5) strongly agree. Cronbach’s **α** = .607. **Direct self- efficacy (n=3):** I am confident/I am sure that I could use an EC in the next month if I wanted to; It is easy for me to use an EC within the next month: (1) strongly disagree – (5) strongly agree. Cronbach’s **α** = .963. |
|  | 18 | **Indirect perceived control:** Control beliefs (N = 9), e.g. *‘the variety of flavours would encourage me to use an e-cigarette*’: (1) unlikely – (5) likely and power of control beliefs (N = 9), e.g. ‘*The variety of flavours makes it’*: (-2) very difficult – (+2) very easy. |
| **4.** Smoking | 2 | Do you currently smoke; Have you ever smoked: yes/no. |
| **5.** Knowledge | 7 | The legal age to purchase an EC is 18 years; EC are cheaper than cigarettes; EC do not contain nicotine; EC do not produce tar and carbon monoxide; EC are addictive; EC are regulated and licensed; EC are 95% less harmful than cigarettes: yes/no. |

**^Note^**^. Indirect TPB items were developed based on the findings from phase 1 (see Table 1). Direct TPB items were developed in line with guidance provided by Ajzen (1991) and Francis et al. (2004) for constructing a TPB-based questionnaire. Knowledge items were taken from the literature. Abbreviations: N = number of items; TPB = Theory of Planned Behaviour; EC = e-cigarette(s).^
